# Data and videos for ultrafast synchrotron X-ray imaging studies of metal solidification under ultrasound

**DOI:** 10.1016/j.dib.2018.01.110

**Published:** 2018-02-08

**Authors:** Bing Wang, Dongyue Tan, Tung Lik Lee, Jia Chuan Khong, Feng Wang, Dmitry Eskin, Thomas Connolley, Kamel Fezzaa, Jiawei Mi

**Affiliations:** a School of Engineering & Computer Science, University of Hull, Hull, HU6 7RX, UK; b Department of Engineering, University of Cambridge, CB2 1PZ, UK; c ISIS Neutron Source, Rutherford Appleton Laboratory, Harwell Oxford, Didcot, OX11 0QX, UK; d Department of Medical Physics and Biomedical Engineering, University College London, London WC1E 6BT, UK; e Brunel Centre for Advanced Solidification Technology, Brunel University London, Uxbridge, London, UB8 3PH, UK; f Diamond Light Source Ltd., Harwell Science & Innovation Campus, Didcot, OX11 0DE, UK; g Advanced Photon Source, Argonne National Laboratory, Argonne, IL 60439, USA

## Abstract

The data presented in this article are related to the paper entitled ‘Ultrafast synchrotron X-ray imaging studies of microstructure fragmentation in solidification under ultrasound’ [Wang et al., Acta Mater. 144 (2018) 505-515]. This data article provides further supporting information and analytical methods, including the data from both experimental and numerical simulation, as well as the Matlab code for processing the X-ray images. Six videos constructed from the processed synchrotron X-ray images are also provided.

**Specifications Table**

**Table:** 

Subject area	*Materials Science and Engineering*
More specific subject area	*Solidification of Metallic Alloys*
Type of data	*Table, figures, synchrotron X-ray images, videos and Matlab code*
How data was acquired	*Synchrotron X-ray imaging, high-speed camera, finite element simulation*
Data format	*Raw and analysed*
Experimental factors	*Ultrasound intensity, acoustic pressure, temperature, fatigue strength and life*
Experimental features	*Solidification of metallic alloys under ultrasound*
Data source location	*School of Engineering & Computer Science, University of Hull, Hull, HU6 7RX, UK*
Data accessibility	*The data are available with this article*

**Value of the data**

• The videos presented clearly demonstrate the microstructural fragmentation induced by ultrasound treatment in a liquid metal.
• The datasets can be used for comparison with other experimental and theoretical results.
• The data on acoustic pressure can be used to determine the ultrasound induced pressure at any depth from the sonotrode tip.
• The Matlab code can be used for future synchrotron X-ray image processing.

## 1 Data

**Fig. 1:**
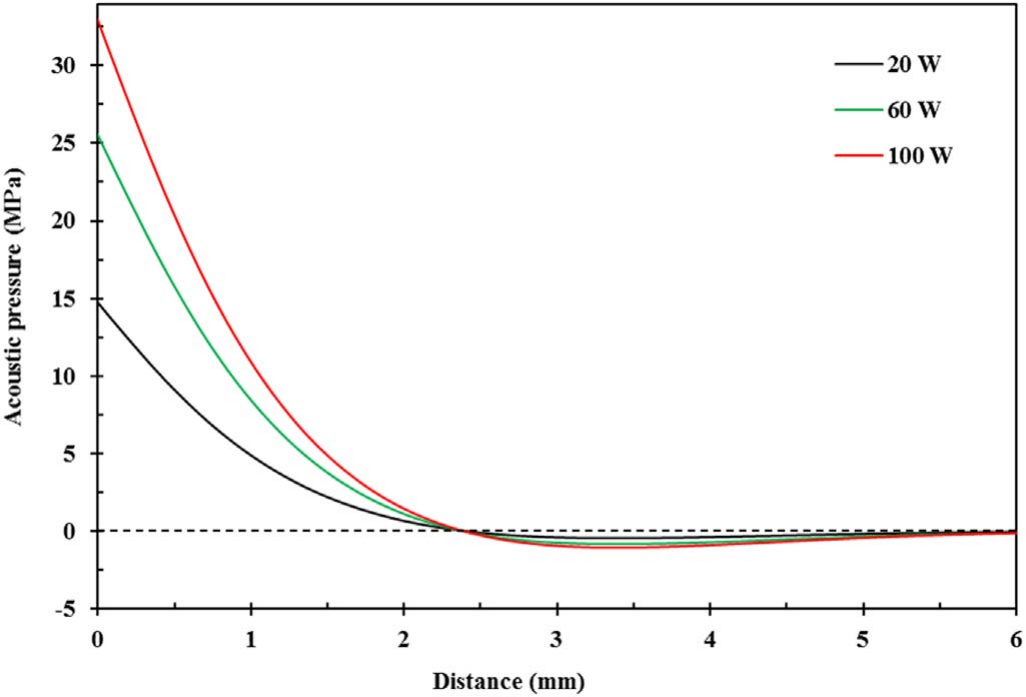
Distribution of the acoustic pressure along the distance below the sonotrode tip under different powers calculated by using Eq. (2).

**Fig. 2:**
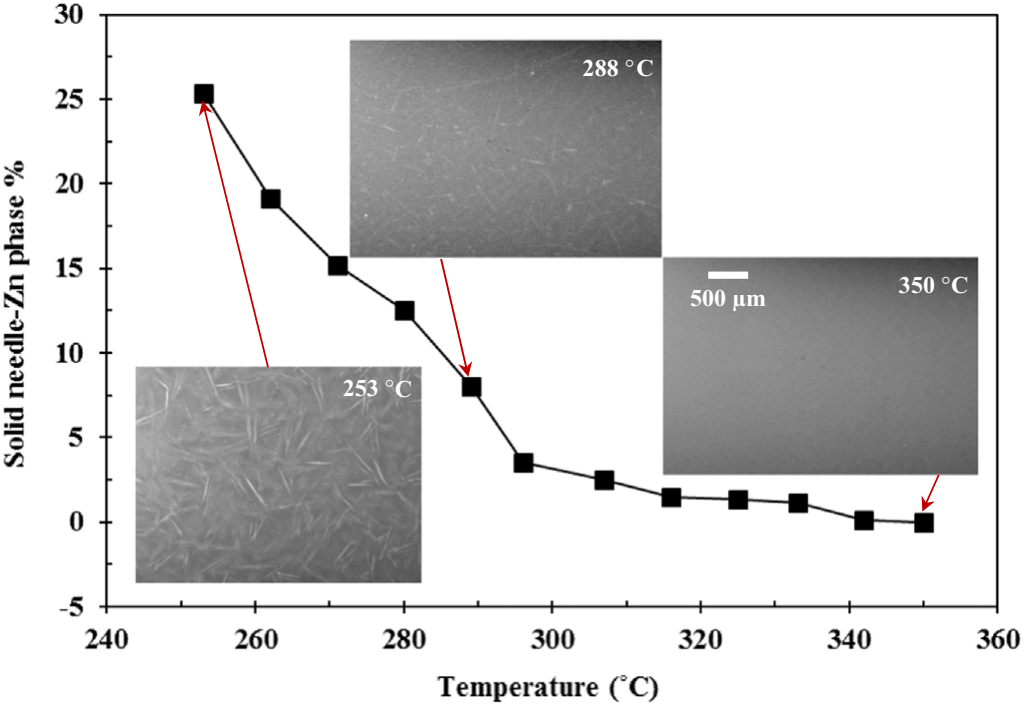
Growth of the solid Zn phase in percentage during the solidification of the Bi-8% Zn alloy under a cooling rate of 0.2 °C/s. Insets show the real-time X-ray images captured at Beamline I12 using 30 fps.

**Fig. 3:**
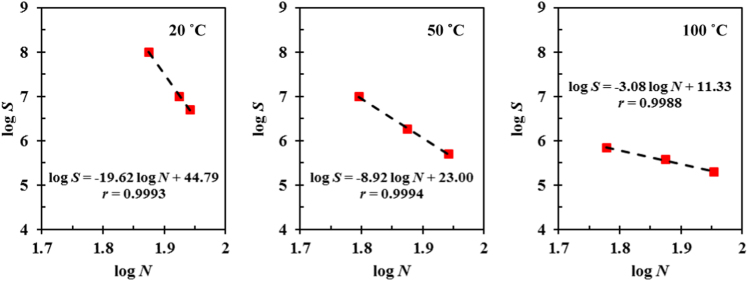
Experimental data extracted to determine the material constants for the Zn alloy at 20, 50 and 100 °C.

Table 1 shows the measured amplitudes of the sonotrode tip from the ultrafast X-ray images when different ultrasound powers were applied; and the resulting ultrasonic intensities. Fig. 1 presents the distribution of acoustic pressure in the Bi-8%Zn liquid metal along the distance below the sonotrode tip. Fig. 2 gives the growth in percentage of solid needle-shaped Zn phase during the solidification process of the Bi-8% Zn alloy without ultrasound under a cooling rate of 0.2 °C/s. Fig. 3 demonstrates the experimental data used to determine the material constants for the Zn alloy at 20, 50 and 100 °C. The Matlab code used to obtain the percentage and speed of the solid particles from synchrotron X-ray images is listed in Appendix A. Synchrotron X-ray images in supporting the evidences and arguments presented in Figs. 4–6 and 8 in [1] were processed into videos. Video 1 shows the ultrasonic bubble implosion and shock wave; Video 2 presents the bubble pulsating on a liquid-solid (L-S) interface; Video 3 gives the fragmentation of a needle-shaped Zn particle by an oscillating bubble; Videos 4–6 demonstrate the break-up of the L-S interface by acoustic flow under different ultrasonic intensities.

**Table 1:** The measured amplitude of the sonotrode tip under different applied ultrasonic powers. The ultrasonic intensity was calculated using Eq. (1).

Ultrasound power (W)	Amplitude (µm)	Ultrasonic intensity (W/mm^2^)
20	30.12	276
40	42.67	553
60	55.22	926
80	65.26	1294
100	77.81	1839

Supplementary material related to this article can be found online at 10.1016/j.dib.2018.01.110.

The following is the Supplementary material related to this article Video 1, Video 2, Video 3, Video 4, Video 5, Video 6.

Video 1 Ultrasonic bubble implosion and shock wave.

Video 2 Ultrasonic bubble pulsating on a liquid-solid interface.

Video 3 Fragmentation of a needle-shaped zinc phase by oscillating bubble.

Video 4 Liquid-solid interface break-up by an acoustic flow (20 W).

Video 5 Liquid-solid interface break-up by an acoustic flow (60 W).

Video 6 Liquid-solid interface break-up by an acoustic flow (100 W)..

## 2 Experimental design, materials and methods

Ultrafast Synchrotron X-ray imaging experiments were carried out to study the effects of ultrasound on the fragmentation of metallic phases during the solidification process as detailed in [1]. A Bi-8%Zn alloy was used because of its low melting temperature and sufficient X-ray contrast between the matrix and the primary Zn phases. A Hielscher UP100H ultrasound processor with a MS2 sonotrode was adopted to introduce ultrasound into the melt. As supplementary to the full research paper [1], this article provides further experimental and simulation data as listed below.

### 2.1 Ultrasonic intensity

To measure the actual vibration amplitudes of the sonotrode tip, the same ultrasound processor as in [1] was used, with the Ø2 mm sonotrode tip submerged in a distilled water tank (50 × 50 × 50 mm). Ultrasound was applied for 1 s, and a Phantom^TM^ V7.3 high speed camera was used to capture the images at 11,000 frame per second (fps) with a spatial resolution of 4.43 µm/pixel. Then, the real vibration amplitudes of the sonotrode tip can be measured from a set of images by using the FIJI [2] software, and the resulting data are listed in Table 1. Therefore, the corresponding ultrasonic intensity, *I*, can be calculated as below [3]:

(1) $$I=\frac{1}{2}\rho C{\left(A_{0}\omega \right)}^{2}$$

where, *ρ* is the density of the melt; *C* is the sound speed in the liquid media; *A*_0_ is the amplitude of the cyclic pressure wave; *ω* = 2π*f* is the angular frequency with *f* being the frequency of the sound wave.

### 2.2 Acoustic pressure

The alternating acoustic pressure field generated from the ultrasonic waves in a liquid medium attenuates exponentially along the distance below the sonotrode tip [4]. The acoustic pressure, *P*_*a*_, at a certain depth follows the Helmoholtz equation [5]:

(2) $$\frac{{\left(\omega /C\right)}^{2}}{\rho }P_{a}+\nabla \left(\frac{1}{\rho }\nabla P_{a}\right)=0$$

Eq. (2) can be solved by using the finite element-based software COMSOL Multiphysics as detailed in [6]. Fig. 1 presents the distribution of acoustic pressure in the Bi-8%Zn liquid metal along the distance below the sonotrode tip under the ultrasonic power of 20 W, 60 W and 100 W.

### 2.3 Solidification of the Bi-8%Zn alloy

Solidification of the Bi-8%Zn alloy without ultrasound was monitored by using the synchrotron X-ray imaging at Beamline I-12, Diamond Light Source (DLS), UK. Fig. 2 shows the growth of the solid needle-shaped Zn phase during the solidification process, the insets are the real-time X-ray images captured at 30 fps.

### 2.4 Fatigue analysis of Zn phases

Experimental data for a commercial Zn-4% Al alloy as reported by Sawalha [7] were used to predict the fatigue behavior of Zn under 270 °C. The fatigue strength, *S*, and fatigue life, *N*, of alloys can be calculated by using the Basquin's Law as detailed in [1]. For high-cycle fatigue (with *N* > 10^5^ cycles), three data points below 90 MPa were selected at each temperature from [7] to determine the material constants. Fig. 3 shows the linear relationship between (log *N*) and (log *C*) at 20, 50 and 100 °C.

### 2.5 Synchrotron X-ray image processing

The percentage and speed of the detached solid particles were obtained by analyzing the X-ray images using a Matlab script [8]. The code is provided in Appendix A.
