# Supplementary material for: Data and videos for ultrafast synchrotron X-ray imaging studies of metal solidification under ultrasound
Source: Data Brief. 2018 Feb 8;17:837–41. doi: 10.1016/j.dib.2018.01.110 (PMC5842321; doi:10.1016/j.dib.2018.01.110)
Supplement: Supplementary file 7 — Supplementary material [file mmc7.docx]

Appendix 1:

The MATLAB script for processing the synchrotron X-ray image datasets in order to determine the positions and trajectories of the particles and bubbles found in liquid metal

clear all

% Input the original image sequences into Matlab

x = which('name of the files');

dirOutput = dir([fileparts(x) filesep 'file name_100*.tif']);

fileNames = {dirOutput.name};

numFrames = numel(fileNames);

I = imread(fileNames{1});

% Convert the original images into binary images

for k = 1:numFrames

Newimage1{k,1}= imread(fileNames{1,k});

Ix=Newimage1{k,1};

BW = im2bw(Ix,0.5);

BW2 = im2uint8(BW);

% Assign different colour to the processed images

BW2=~BW2;

BW2 = bwareaopen(BW2, 4);

BW3{k,1}=BW2;

end

for k = 1:numFrames

Ix=Newimage1{k,1};

% Fill image holes

BW4 = imfill(BW3{k,1},'holes');

% Search and find the centre position of the particles

s = regionprops(BW4, 'centroid');

centroids{k} = cat(1, s.Centroid);

c{k} = struct2cell(s);

imshow(Ix);

hold(imgca,'on');

plot(imgca, centroids{k}(:,1), centroids{k}(:,2), 'ro','MarkerFaceColor', 'r', 'MarkerSize',3);

hold(imgca,'off');

saveas(imgca,sprintf('Processed Image # %d.tif',k));

end
