# Supplementary material for: Data and videos for ultrafast synchrotron X-ray imaging studies of metal solidification under ultrasound
Source: Data Brief. 2018 Feb 8;17:837–41. doi: 10.1016/j.dib.2018.01.110 (PMC5842321; doi:10.1016/j.dib.2018.01.110)
Supplement: Supplementary file 8 — Transparency document [file mmc8.docx]

Conflict of Interest

The author declares no Conflict of Interest.
